# Innovative Multi-View Strategies for AI-Assisted Breast Cancer Detection in Mammography

**DOI:** 10.3390/jimaging11080247

**Published:** 2025-07-22

**Authors:** Beibit Abdikenov, Tomiris Zhaksylyk, Aruzhan Imasheva, Yerzhan Orazayev, Temirlan Karibekov

**Affiliations:** Science and Innovation Center “Artificial Intelligence”, Astana IT University, Astana 010000, Kazakhstan; aruzhan.imasheva@astanait.edu.kz (A.I.); y.orazayev@astanait.edu.kz (Y.O.); t.karibekov@astanait.edu.kz (T.K.)

**Keywords:** deep learning, mammography, breast cancer, computer-aided diagnosis (CADx), medical image analysis

## Abstract

Mammography is the main method for early detection of breast cancer, which is still a major global health concern. However, inter-reader variability and the inherent difficulty of interpreting subtle radiographic features frequently limit the accuracy of diagnosis. A thorough assessment of deep convolutional neural networks (CNNs) for automated mammogram classification is presented in this work, along with the introduction of two innovative multi-view integration techniques: Dual-Branch Ensemble (DBE) and Merged Dual-View (MDV). By setting aside two datasets for out-of-sample testing, we evaluate the generalizability of the model using six different mammography datasets that represent various populations and imaging systems. We compare a number of cutting-edge architectures on both individual and combined datasets, including ResNet, DenseNet, EfficientNet, MobileNet, Vision Transformers, and VGG19. Both MDV and DBE strategies improve classification performance, according to experimental results. VGG19 and DenseNet both obtained high ROC AUC scores of 0.9051 and 0.7960 under the MDV approach. DenseNet demonstrated strong performance in the DBE setting, achieving a ROC AUC of 0.8033, while ResNet50 recorded a ROC AUC of 0.8042. These enhancements demonstrate how beneficial multi-view fusion is for boosting model robustness. The impact of domain shift is further highlighted by generalization tests, which emphasize the need for diverse datasets in training. These results offer practical advice for improving CNN architectures and integration tactics, which will aid in the creation of trustworthy, broadly applicable AI-assisted breast cancer screening tools.

## 1. Introduction

Breast cancer remains a significant global health issue, impacting one in eight women during their lifetime. Notwithstanding decades of advancements in screening and therapy, early diagnosis continues to be the most effective means of decreasing death. Mammography, a low-dose X-ray imaging technique, has historically served as the primary method for screening [1]. Nonetheless, its diagnostic utility possesses certain limitations. The interpretation of mammograms is a complex and demanding endeavor, frequently affected by breast density, imaging abnormalities, and subtle radiographic indicators that may be overlooked, particularly in the initial stages of malignancy. Furthermore, even seasoned radiologists are prone to inter-reader variability, and error rates are significant [2].

In recent years, deep learning (DL) has become a revolutionary breakthrough in medical imaging. Deep learning, especially convolutional neural networks (CNNs), demonstrates significant potential in tasks including tumor detection, segmentation, and classification due to its capacity to generate hierarchical representations from unprocessed input [3]. Encouraged by its success in natural image classification, researchers have turned to CNNs for automating breast cancer detection from mammographic images. Yet, despite encouraging performance reported in controlled research settings, clinical deployment remains elusive. The question is not whether these models can learn, but whether they can generalize.

A persistent problem in the literature is the excessive dependence on single-dataset evaluation. Models that are trained and evaluated on a singular mammography dataset frequently exhibit remarkable performance; nonetheless, they falter when utilized with data from disparate institutions, imaging modalities, or patient demographics. This phenomenon, referred to as domain shift, reveals the vulnerability of numerous deep learning models and emphasizes the necessity of developing systems that are resilient across diverse clinical environments [4].

A further neglected difficulty in contemporary methodologies is the restricted utilization of contextual anatomical data. In practice, radiologists do not assess a singular mammographic view in isolation [5]. Each breast is generally scanned from two standard angles: the craniocaudal (CC) and the mediolateral oblique (MLO) views. These contrasting perspectives elucidate distinct facets of breast anatomy and lesion shape, with the diagnostic procedure predominantly dependent on the integration of information from both sources. Many CNN-based classifiers, however, regard these views as independent inputs, overlooking the potential to replicate this multi-perspective reasoning.

This study originated from two principal observations: first, that model performance must be evaluated across varied datasets to guarantee clinical applicability; and second, that a more sophisticated integration of CC and MLO views could yield enhanced representations for classification. Consequently, we undertook a thorough analysis with the following objectives:

- To evaluate various transfer learning models across diverse mammographic data sources, determining the most effective CNN architectures for reliable breast cancer classification;
- To properly assess generalizability, models should be trained on a subset of datasets while reserving others exclusively for testing, therefore imitating real-world deployment where data distribution may markedly differ from training settings;
- To propose and assess two innovative strategies for dual-view integration: one that consolidates CC and MLO views into a singular image representation and another that employs a dual-branch CNN architecture, enabling the model to acquire specialized features from each view prior to joint inference.

We assembled six mammographic datasets obtained from various demographics and imaging devices, reflecting the diversity encountered in global clinical applications. This study seeks to advance beyond controlled benchmarks by integrating architectural comparisons, view integration methodologies, and comprehensive cross-dataset evaluations, ultimately aiming to build generalizable, interpretable, and clinically relevant AI tools for breast cancer screening.

The rest of the paper is organized as follows. In Section 2, related work on breast cancer classification is discussed. The proposed methodology is presented in Section 3. In Section 4, the obtained experimental results are presented. Section 5 discusses the obtained results. Finally, Section 6 concludes the work.

## 2. Related Work

In the dynamically changing world of breast cancer detection, mammography is still the gold standard for early detection. However, the nuances in the manifestation of lesions call for analytical acumen that can be exceeded by the human eye. The following is a review of the timeline of mammogram image classification, the turning points at which convolutional neural networks (CNNs), vision transformers (ViTs), and the use of single-view versus multi-view (CC and MLO) strategies all play a critical part.

One of the methods used for mammography image classification involves pairing the classification process with the detection of lesions. Salama and Aly utilized a two-stage pipeline, where the first stage performs the segmentation using a modified U-Net model, and the following stage classifies the segmented mask with pre-trained models [6]. Rehman et al. utilized a similar method, identifying architectural distortion, then classifying it into benign or malignant [7]. Ribli et al. utilized faster R-CNN for combined lesion localization and classification [8]. Another study took a different approach, by initially classifying full mammogram with CNN, then localizing the tumors [9].

Other studies have explored CNN-based feature extraction as a key component of the classification pipeline. Kooi et al. merged CNN feature extraction with traditional CAD pipelines to outperform handcrafted techniques [10]. Haq et al. (2022) proposed a deep learning-based CAD system for mammogram classification that combines feature fusion and ensemble learning in a custom CNN [11]. Two fusion strategies (with and without 1 × 1 convolutions) are applied to combine features from different CNN layers. Classification is performed using three classifiers—dense layer, SVM, and random forest—merged via majority voting. Another similar study by Chakravarthy et al. used a hybrid approach that fused features from multiple CNNs—VGG16, VGG19, ResNet50, and DenseNet121—into a single classification pipeline [12]. Samee et al. implement pre-trained CNN models for feature extraction with univariate-based feature selection approach to overcome feature dimensionality curse (FDC) and multicollinearity [13]. Zahoor et al. proposed a distinctive hybrid framework integrating the use of the MobileNet and NasNet feature extractors with a nature-inspired optimization algorithm known as the whale optimization algorithm (MEWOA) [14]. Their feature-optimized model reached a maximum 99.8% accuracy on the MIAS, INbreast, and CBIS-DDSM databases.

Another study implemented a weakly supervised, attention-based deep learning system for breast cancer classification using full-field digital mammograms [15]. Their model, based on clustering-constrained attention multiple instance learning (CLAM), processes high-resolution CC and MLO images as patch sets and uses attention pooling to focus on the most informative regions. By combining CC and MLO views, the multimodal setup improved performance, achieving an AUC-ROC of 0.896 and F1-score of 81.8% on the MUG dataset. The model also offers explainability through attention maps and generalizes well on external datasets like CMMD.

Several of them extended boundaries further. StethoNet introduced a CNN ensemble model for full-field mammogram classification based on view-specific voting techniques and entropy-based normalization [16]. It performed better than most ROI-based schemes across the CMMD, INbreast, and Vindr-Mammo databases without requiring tuning specifics for datasets. Elkorany et al. created a CNN-based system with a combination of feature extraction using term variance and multiclass SVM, with maximum accuracy for the MIAS database [17].

Transformer models also have shown promise for full-image analysis. Ayana et al. compared ViT, Swin Transformer, and PVT models against the DDSM benchmark, all registering perfect classification when pretrained [18]. Training from scratch for the models caused performance degradation, further affirming the valuable contribution of transfer learning in medical imaging.

Building on the individual strengths of CNNs, which excel at capturing local spatial features, and transformers, which effectively model long-range dependencies and global context, CNN–Transformer hybrid approaches combine these complementary capabilities to enhance performance in complex tasks like mammogram classification, seamlessly integrating detailed local analysis with comprehensive temporal and contextual understanding. Despite the achievements of standalone CNN and transformer-based methods, Jeny et al. introduced a CNN–Transformer-based model for breast cancer classification using mammography, integrating both prior and current images to detect temporal changes, mimicking radiologists’ diagnostic processes [19]. The model combines a modified ResNet50 for feature extraction, positional encoding, transformer encoder blocks with multi-head self-attention, and channel attention to enhance feature representation, achieving an accuracy of 90.80% and an AUC of 92.58% on the UCHC dataset. By employing focal loss, it effectively reduces false positives and negatives, outperforming baseline models like ResNet50, Siamese networks, and others in detecting abnormalities such as masses, calcifications, and architectural distortions. Future work aims to extend the model to include benign cases and integrate multi-view analysis for improved diagnostic accuracy.

In spite of the success of single-view models, clinical application is necessarily based on multiple views: usually CC and MLO for each breast, i.e., four per patient. Building off of the aforementioned, Patheda et al. presents a hybrid deep learning model that integrates convolutional neural networks (CNNs) with vision transformers (ViTs). The CNN component captures fine-grained local features, while the ViT processes global contextual information, enabling more accurate differentiation between benign and malignant cases. Evaluated on a balanced dataset of 10,000 images, the model achieved 90.1% validation accuracy with minimal overfitting. Compared to conventional and pre-trained models, the hybrid approach demonstrated a strong balance between accuracy and generalization, making it promising for clinical applications. They proposed a CLAHE preprocessing-enabled CNN-ViT architecture for increasing the contrast in dense breast tissue [20].

An innovative GAN-based method by Yamazaki and Ishida suggested generating missing views (e.g., CC from MLO) with CR-GANs. Tested with INbreast, CMMD, and CBIS-DDSM, their model constructed synthetic views with high fidelity, reducing the issue of scarcity of data in practical applications [21]. Large-scale validation procedures also had a critical impact. Medjeded et al. introduces a novel triplet convolutional neural network (CNN) to classify breast lesions in mammograms as benign or malignant, using three inputs: the region of interest (ROI), its Canny-filtered version, and the whole mammogram [22]. Tested on an augmented DDSM dataset of 4000 images, the model achieved an accuracy of 93.13%, with 96% sensitivity and 90.25% specificity. Finally, Hussain et al. introduced a novel “Multiview Multimodal Feature Fusion (MMFF)” approach to enhance breast cancer classification. It combines four standard mammographic views (Left/Right CC and MLO) with structured metadata from radiology reports, including age, breast density, BIRADS scores, family history, and lesion laterality [23]. The team curated an in-house dataset from TecSalud Hospitals in Monterrey, Mexico, comprising 3080 images from 770 cases. Mammograms were converted from DICOM to TIFF, resized, and augmented to boost model robustness. Image features were extracted using a ResNet50 with Squeeze-and-Excitation (SE) blocks, while an ANN processed the textual metadata. A late fusion strategy merged both modalities before classification via another ANN. The MMFF model significantly outperformed single-modal approaches, achieving 96.9% accuracy, 97.7% precision, 91.6% sensitivity, and a 96.5% AUC, highlighting the promise of multimodal integration in medical diagnostics.

Several earlier studies have explored multi-view fusion in mammography, including StethoNet [16], which applies entropy-based voting on view-specific predictions; MMFF, which combines four views and radiological metadata using a late fusion ANN; and CLAM, which utilizes attention-based pooling on patch-level features from CC and MLO views. While effective, these methods often integrate views in complex or task-specific ways, making it difficult to isolate the contribution of view fusion itself. In contrast, our work introduces and compares two generalizable, architecture-agnostic fusion strategies—MDV and DBE—allowing a direct comparison between early and late fusion paradigms under consistent experimental conditions across multiple datasets.

## 3. Materials and Methods

### 3.1. Datasets

**Figure 1:**
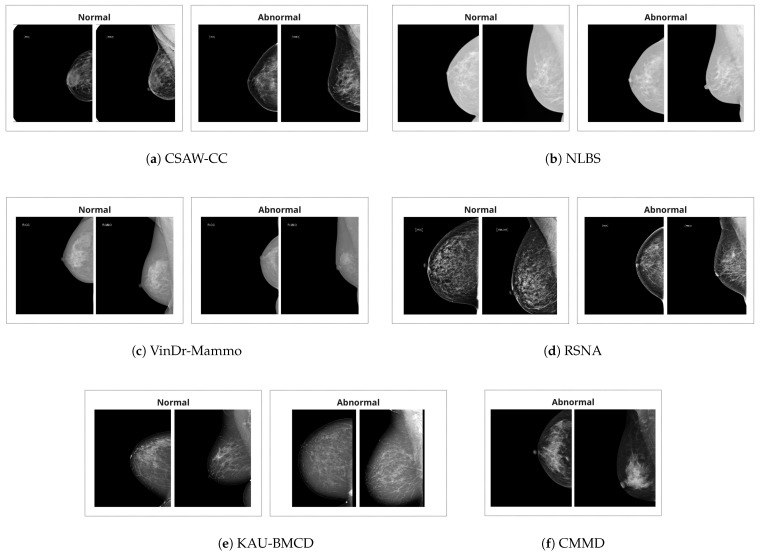
Example craniocaudal (CC) and mediolateral oblique (MLO) view pairs of one normal breast and one breast with an abnormality, taken from various mammography datasets. In each pair, the CC view is shown on the left and the MLO view on the right.

There are a total of six datasets utilized in this study. The summary of the datasets is provided in Table 1. Additionally, example images are provided in Figure 1.

VinDr-Mammo [24] is a large-scale FFDM dataset curated from hospitals in Vietnam. It consists of 5000 exams (20,000 images), with breast-level labels following BI-RADS categories. All exams were double-read and reconciled by a third radiologist in cases of disagreement. The dataset provides rich metadata and preserves key DICOM fields while ensuring patient anonymity.

CMMD (Chinese Mammography Database) [25] includes 3734 images from 1775 patients, all of which are abnormal. This dataset is unique in that it includes biopsy-confirmed tumor types and molecular subtypes for a subset of cases, making it suitable for diagnostic model development.

CSAW-CC (Swedish Mammography Dataset) [26] was collected for AI-based breast cancer research and includes over 82,000 images from Karolinska University Hospital. The dataset contains both cases and healthy controls with annotations at the pixel level, along with rich metadata such as histology, tumor size, and lymph node involvement. All normal cases were verified as cancer-free for at least two years.

NLBS (NL Breast Screening Dataset) [27] offers high-resolution DICOM mammograms from over 8000 patients. With more than 26,000 images, the dataset includes normal, false positive, and biopsy-confirmed cancer cases. It closely mirrors real-world screening distributions and provides sufficient data for training and evaluating models under clinical class imbalance.

KAU-BCMD [28] was compiled at King Abdulaziz University in Saudi Arabia. The Kaggle-available subset used here consists of 2180 images with BI-RADS categories determined via consensus of three experts. Despite class imbalance (majority BIRADS 2), the dataset includes both benign and suspicious cases across multiple BI-RADS levels.

RSNA Breast Cancer Detection Dataset [29] is sourced from a recent Kaggle competition and includes more than 47,000 images from multiple institutions. Although metadata is partially available only for training data, the dataset supports large-scale supervised learning and cross-site evaluation. Images are provided in DICOM format, and labels include cancer status, density, and biopsy outcomes.

To address class imbalance during training while preserving the overall distributional characteristics of each dataset, we applied balanced undersampling. Specifically, the number of normal cases in the training sets was reduced so that the proportion between normal and abnormal samples remained consistent with that of the corresponding test sets. This strategy ensures that the datasets remain representative of their original class distributions, while reducing the risk of model bias toward the majority class. The resulting sample counts are summarized in Table 2.

### 3.2. Combining Two Mammogram Views

In this study, we explore two distinct strategies to incorporate the most common mammographic views (CC and MLO) into the classification framework. These views are routinely acquired in clinical mammography, offering complementary anatomical perspectives critical for an accurate diagnosis. Since the diagnostic value of integrating both views is high, we designed and evaluated two methodological pipelines to leverage this dual-view information: Merged Dual-View (MDV) and Dual-Branch Ensemble (DBE).

Both strategies share a common preprocessing pipeline for individual mammographic images. First, we perform breast region detection to isolate the breast region and eliminate irrelevant structures such as labels or artifacts.

To eliminate irrelevant structures such as black borders and machine labels, we scanned each image vertically from the top and bottom edges toward the center, checking pixel columns for black content. The scan continued until a column was encountered that contained at least one non-black pixel. To ensure no useful tissue information was inadvertently excluded, we added a safety margin by shifting the cropping boundary five pixels back from this point. The final crop was applied based on these adjusted boundaries, helping to remove black padding while preserving relevant breast tissue.

To correct anatomical orientation, we applied horizontal flipping based on laterality: left breast (L) images were flipped such that all inputs maintain consistent left-right alignment.

Finally, to create a combined representation for the Merged Dual-View (MDV) method, preprocessed CC and MLO views of the same breast were concatenated side by side into a single RGB image. Each view underwent identical processing and was placed to preserve anatomical consistency. The combined image was normalized using [mean = 0.5, std = 0.5] and converted to a PyTorch 2.6.0 tensor.

A quality-control step was implemented to exclude any images with loading errors or missing views, and we ensured that brightness inversions (e.g., white background instead of black) were corrected automatically based on global intensity thresholds (images with mean intensity > 127 were inverted).

**Figure 2:**
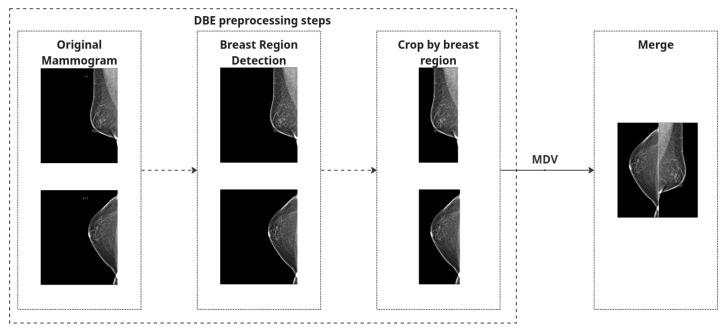
Preprocessing process.

**Figure 3:**
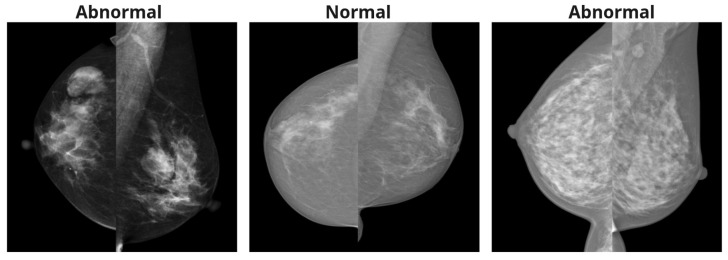
Mammography images after processing for the MDV method.

These preprocessing steps are applied independently to both CC and MLO images. For the MDV method, the views are combined, and Figure 2 represents preprocessing steps for both approaches. Figure 3 shows the examples of mammography images for the MDV approach.

**Figure 4:**
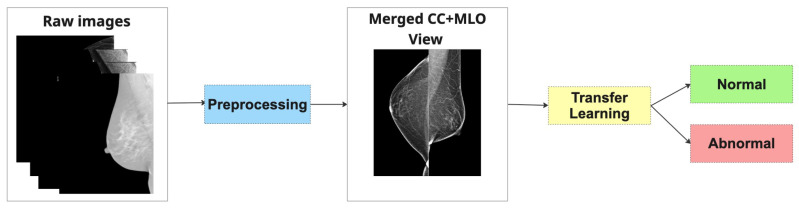
Pipeline of Merged Dual-View (MDV).

**Figure 5:**
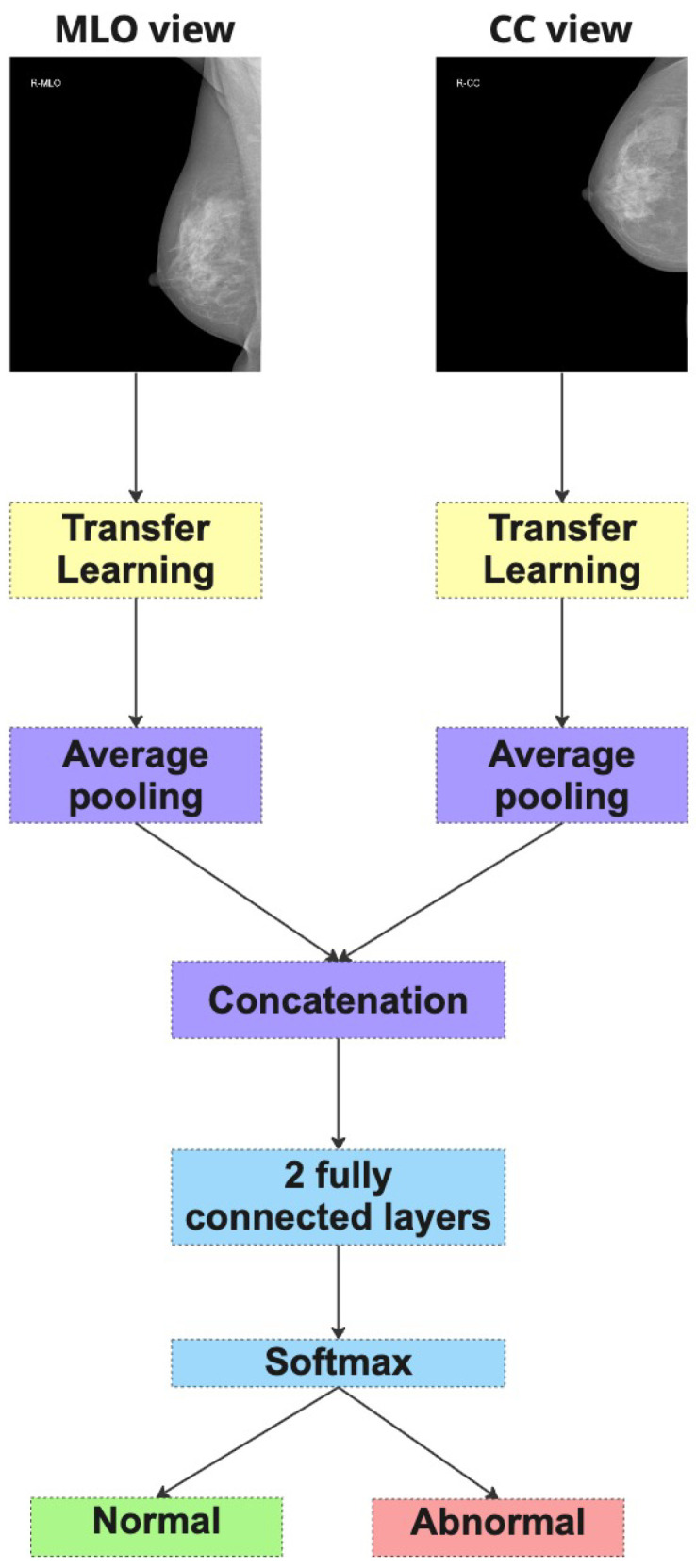
Dual-Branch Ensemble (DBE).

- **Method 1: Merged Dual-View (MDV)**
  In this strategy, the CC and MLO views of the same breast are spatially fused into a single composite image prior to being input into the classification model. Specifically, the images are concatenated—side by side, without overlap—forming a unified visual representation that encapsulates both anatomical perspectives (as depicted in Figure 4). This merged image is treated as a standard input for a single convolutional neural network (CNN), which is then trained to extract joint features and perform classification. The underlying hypothesis is that by presenting both views concurrently, the model can learn richer, more discriminative representations that capture inter-view correlations relevant to malignancy.
- **Method 2: Dual-Branch Ensemble (DBE)**
  The second strategy follows a more modular design. Here, two independent CNN models are trained in parallel—one dedicated to the CC view and the other to the MLO view (as depicted in Figure 5). Each model learns to extract features specific to its respective projection. During inference, both models produce separate probability predictions for the same case, which are then averaged to yield a final classification score. This ensemble-based approach allows each model to specialize in its view-specific anatomical characteristics while combining their complementary outputs to make a consensus decision. The DBE method is particularly advantageous in scenarios where the learning dynamics or visual features of each view differ significantly.

Both strategies aim to emulate the radiologist’s natural diagnostic workflow, where both views are considered together to improve diagnostic accuracy. In subsequent sections, we compare the performance of these two methods across multiple architectures and datasets to evaluate their relative strengths and generalization capabilities.

### 3.3. Transfer Learning

Transfer learning has become a cornerstone in medical imaging tasks, especially in domains like mammography where labeled data is scarce and training deep neural networks from scratch is computationally expensive and often impractical. By leveraging pretrained models originally trained on large-scale natural image datasets (e.g., ImageNet), transfer learning enables the reuse of generic visual feature representations that can be fine-tuned for more specialized medical tasks. In this study, we adopted transfer learning as the backbone of our classification framework and systematically evaluated a suite of state-of-the-art architectures.

We investigated the performance of several well-established deep learning models, including ResNet-18 [30], ResNet-50 [30], EfficientNet-B0 [31], DenseNet [32], Inception [33], MobileNet [34], and Vision Transformer (ViT-B/16) [35]. These models were fine-tuned on our mammographic datasets using both the Merged Dual-View (MDV) and Dual-Branch Ensemble (DBE) strategies described earlier.

### 3.4. Experimentation Settings

To evaluate the generalization and robustness of our proposed models, we designed two distinct experimental settings:

**1. Generalization Evaluation (Seen vs. Unseen):**

In this setup, we assess the model’s ability to generalize to unseen data using a leave-one-dataset-out approach. Specifically, we train on five out of six available datasets and test on the remaining one, ensuring that each dataset is used once as the held-out test set, except for CMMD, which is excluded from isolation due to containing only one class. This constitutes the *unseen* condition, where the test dataset is entirely excluded from training to evaluate true generalization.

For comparison, in the *seen* condition, the target dataset is included in the training set; however, the test samples are drawn from a disjoint subset—i.e., we apply a within-dataset split (typically 80/20), ensuring no sample leakage. This comparison enables us to quantify how performance is affected when the model has seen data from the test distribution during training versus when it has not.

**Figure 6:**
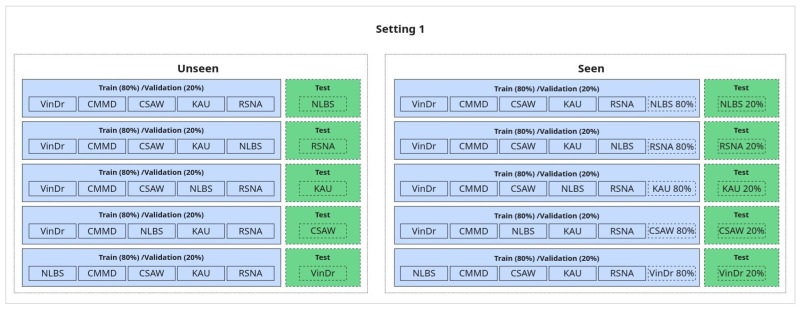
Generalization setup: seen vs. unseen.

An illustration of this setup is shown in Figure 6.

**2. Refined Evaluation with Selected Datasets (80/20 Split):**

**Figure 7:**
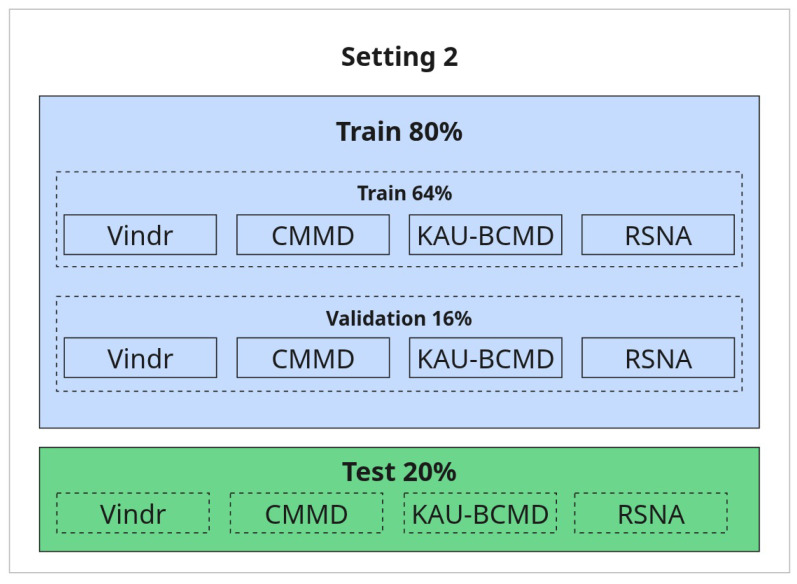
Refined setup with 80/20 train-test split.

In this setting, a subset of datasets was selected for training and testing using a standard 80/20 split. All datasets used in this setup are included in both training and testing, ensuring the test samples come from a known distribution. The rationale for selecting these datasets and excluding others is discussed in detail in the Results section. The illustration is provided in Figure 7.

### 3.5. Evaluation Metrics

To evaluate the performance of the classification models, we utilized a set of widely adopted metrics in binary classification, particularly suited for imbalanced datasets such as mammographic image analysis. These metrics provide a well-rounded understanding of the model’s predictive behavior in both seen and unseen scenarios. All metrics are reported for the positive class (i.e., cancer). This is consistent with clinical priorities, where correctly identifying cancerous cases is of critical importance.

- **Accuracy**:
  Accuracy is the proportion of correctly classified instances among the total number of samples. While straightforward, it can be misleading in imbalanced datasets and should be interpreted with care.
  (1) $$\mathrm{Accuracy}=\frac{TP+TN}{TP+TN+FP+FN}$$
- **Precision (Positive Predictive Value)**:
  Precision measures the proportion of true positive predictions among all positive predictions. High precision is essential in reducing false positives, which is critical in clinical diagnostics to avoid unnecessary biopsies or interventions.
  (2) $$\mathrm{Precision}=\frac{TP}{TP+FP}$$
- **Recall (Sensitivity)**:
  Recall represents the proportion of actual positives that are correctly identified by the model. In the context of breast cancer detection, recall is a key metric, as failing to identify a malignant case can have serious consequences.
  (3) $$\mathrm{Recall}=\frac{TP}{TP+FN}$$
- **F1-Score**:
  The F1-score is the harmonic mean of precision and recall. It is a balanced metric that is particularly informative when dealing with class imbalance.
  (4) $$F1\;\mathrm{-Score}=2\times \frac{\mathrm{Precision}\times \mathrm{Recall}}{\mathrm{Precision}+\mathrm{Recall}}$$
- **AUC-ROC (Area Under the Receiver Operating Characteristic Curve)**:
  AUC-ROC evaluates the model’s ability to discriminate between the two classes across all classification thresholds. It is a threshold-independent metric that reflects overall classification performance, especially useful when comparing models.

The combination of threshold-dependent metrics (e.g., accuracy, precision, recall, F1) and the threshold-independent AUC-ROC allows for a comprehensive evaluation of model performance. This is particularly important for ensuring the reliability of AI systems in real-world clinical deployment.

### 3.6. Implementation Details

All experiments were conducted on a local workstation equipped with dual **NVIDIA GeForce RTX 2080 Ti** GPUs (Santa Clara, CA, United States), each with 11 GB of VRAM. GPU acceleration was enabled via CUDA and cuDNN, and the software environment was built on Ubuntu 20.04, with Python 3.9 and PyTorch 2.6.0 as the primary deep learning framework.

Model training and evaluation were implemented in PyTorch, utilizing its modular API for flexible model design and efficient data handling. Training was parallelized using DataParallel, allowing simultaneous utilization of both GPUs.

The key implementation parameters were as follows:

- **Batch size:** 32 (adjusted based on available GPU memory);
- **Optimizer:** Adam ($\beta_{1}=0.9$, $\beta_{2}=0.999$);
- **Initial learning rate:** $1\times 10^{-4}$ with cosine annealing schedule;
- **Loss function:** Binary Cross-Entropy Loss;
- **Epochs:** 30–50, selected based on convergence behavior observed in preliminary experiments; early stopping was applied based on validation loss. Specifically, 30 epochs were used for experiments involving a single dataset training, while 50 epochs was applied in cross-dataset settings;
- **Early stopping:** Applied with a patience of 5 epochs, based on validation loss;
- **Input size:** All images were resized to 224 × 224 pixels. For the DBE approach, CC and MLO views were independently resized. For the MDV approach, views were first concatenated side by side into a wider image, which was then resized to 224 × 224.

To ensure consistent evaluation and reproducibility, the same preprocessing pipeline and training protocol were applied across all models and datasets. Random seeds were fixed for all data splits and model initializations. Model performance was monitored using TensorBoard, where training loss, validation AUC, and confusion matrices were tracked in real time.

This hardware and software setup enabled us to efficiently conduct a comprehensive series of experiments, including architectural comparisons, cross-dataset evaluation, and dual-view integration strategies, within a practical time frame.

## 4. Results

### 4.1. Generalization Evaluation: Seen vs. Unseen Dataset Settings

This subsection provides a detailed analysis of the generalization behavior of both MDV-based and DBE-based model architectures under two evaluation conditions: seen and unseen, as defined in Setting 1 (Figure 6). Table 3, Table 4, Table 5 and Table 6 present the classification performance across multiple datasets using standard metrics.

Across all models, performance in the seen condition was consistently higher than in the unseen condition. For instance, ResNet18 (MDV) achieved a recall of 0.6543 and ROC AUC of 0.6197 on CSAW-CC in the seen setting, which dropped to 0.6842 recall but a much lower 0.5674 ROC AUC in the unseen setting—indicating poorer discriminative performance despite recovering some sensitivity.

More notably, models trained under the DBE setup showed improved seen performance across almost all metrics compared to MDV models. For example, on the NLBS dataset, ResNet18 (DBE) achieved 0.5400 accuracy, 0.5665 F1, and 0.5695 ROC AUC, clearly outperforming the MDV counterpart. However, in the unseen condition, even the DBE model struggled—e.g., recall dropped to 0.1427 on NLBS and 0.0674 on KAU-BCMD, indicating severe generalization gaps.

Precision values were generally modest across both setups and conditions. Despite some high-precision values in the seen setting (e.g., ResNet18 (MDV) on VinDr-Mammo: 0.6937), these did not translate well to the unseen setting. For example, ResNet18 (DBE) precision reached 0.7407 on VinDr-Mammo, but recall collapsed to 0.0243, leading to an ineffective overall F1 score of just 0.0470.

While Inception (MDV) showed high accuracy on unseen NLBS (0.8287), the recall (0.1776) and F1 (0.2390) were still low, confirming that the model may favor the majority class. This highlights the challenge of class imbalance in mammography classification and questions the usefulness of accuracy alone as a metric.

Overall, recall was consistently better in seen settings across all models and datasets, while unseen recall remained poor, suggesting that deep learning models, even under the DBE structure, are still heavily dependent on the distribution of training data. This reinforces the concern that such models may overfit to dataset-specific features and fail to generalize across imaging domains.

The results underline a clear generalization gap between seen and unseen patient conditions, revealing that visual variability and domain shift continue to pose major challenges. Furthermore, the overall low F1 scores and inconsistent ROC AUCs across datasets emphasize the importance of robust cross-domain learning strategies. Without addressing these limitations, diagnostic models are unlikely to perform reliably across diverse clinical settings and patient populations.

### 4.2. Dataset-Specific Performance Analysis

After the evaluation of generalization of the models, a dataset-level performance analysis was conducted to understand how individual datasets influence the behavior of the Merged Dual-View (MDV) and Dual-Branch Ensemble (DBE) models. Each model was trained and tested on a single dataset independently. CMMD was excluded due to the lack of normal cases. This analysis serves to uncover potential dataset-specific biases, varying levels of difficulty, and their contribution to decreased generalization when combined in multi-source training.

**Figure 8:**
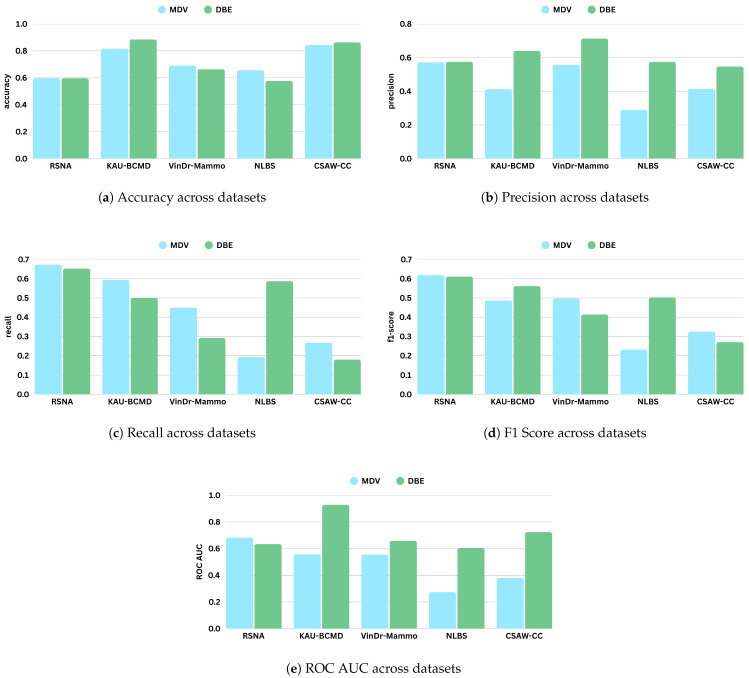
Comparison of evaluation metrics (accuracy, precision, recall, F1 score, and ROC AUC) for ResNet18 across different mammography datasets under Merged Dual-View (MDV) and Dual-Branch Ensemble (DBE) frameworks. Each subfigure presents a bar chart for a specific metric.

Table 7 and Table 8 present the performance metrics of the MDV and DBE models across five mammography datasets: RSNA, KAU-BCMD, VinDr, NLBS, and CSAW-CC. Figure 8 shows the comparative digrams for each metric across datasets and methods.

The performance varied across the datasets for the MDV model. The model achieved its highest accuracy on CSAW-CC and KAU-BCMD. However, the precision and the recal scores remain low, indicating that the accuracy is probably inflated due to class imbalance, with the model possibly favoring majority classes.

NLBS appeared to be the most challenging dataset. With the MDV model, it showed the lowest F1 score (0.2315) and ROC AUC (0.2740), pointing to both poor classification and weak class separability. The low precision (0.2898) and recall (0.1927) underscore a general inability to detect minority or complex cases within NLBS, potentially because of domain-specific image characteristics, such as lower contrast, unique noise profiles, or variations in annotation.

VinDr exhibited intermediate performance with the MDV architecture, achieving an accuracy of 0.691, and a modest F1 score of 0.4984. The recall of 0.4501 suggests some sensitivity to positive cases, though precision (0.5582) and ROC AUC (0.5555) indicate only limited discriminative power.

On RSNA, the DBE and MDV models performed similarly in accuracy (0.5967), but the DBE model achieved a slightly better F1 score (0.6111 vs. 0.6187) and a more balanced precision–recall tradeoff, reflecting a more robust output distribution.

Most notably, DBE significantly outperformed MDV on the KAU-BCMD dataset. The accuracy rose to 88.53%, with a remarkably high ROC AUC of 0.9283. These results suggest that the DBE architecture may better exploit inter-view feature complementarities or capture latent patterns in datasets.

For VinDr, the DBE model reported mixed results: while it achieved high precision (0.7127), the recall dropped to 0.2921, resulting in a moderate F1 score of 0.4144.

Interestingly, the DBE model performed notably better than MDV on NLBS, where it achieved an F1 score of 0.5808 and a ROC AUC of 0.6050. Compared to the MDV results on NLBS, this marks a considerable improvement, suggesting that the ensemble structure may enhance generalizability or robustness to the noisier or more heterogeneous image characteristics present in NLBS.

On CSAW-CC, both models reported high accuracy (over 84%). However, the recall remained low (0.1801), leading to a modest F1 score of 0.2709. This again reflects the tendency of both models to favor dominant classes, especially in datasets with imbalanced class distributions.

**Figure 9:**
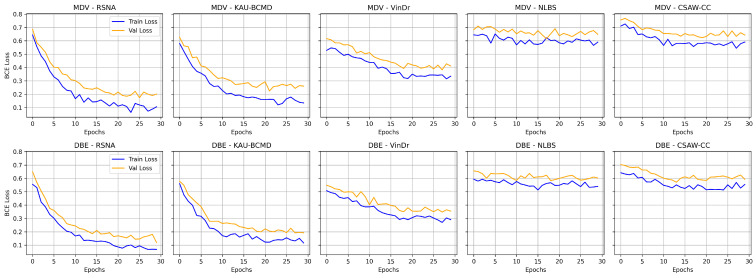
Training vs. validation loss for MDV and DBE models across datasets.

Overall, the dataset-specific analysis reveals substantial variability in model performance across domains. Figure 9 illustrates the training and validation loss curves for the MDV and DBE models across multiple mammography datasets. NLBS and CSAW-CC consistently emerge as difficult datasets, suggesting they may act as a limiting factor in joint training scenarios. In contrast, datasets like KAU-BCMD exhibit more stable and predictable model behavior, particularly under the DBE architecture. These findings underscore the importance of dataset selection, data quality assessment, and tailored domain adaptation strategies in future work to develop more reliable and generalizable mammography classification models.

### 4.3. Evaluating Performance After Excluding Low-Performing Datasets

Based on the dataset-specific performance analysis, the training configuration was refined, which excluded two datasets (NLBS and CSAW-CC) that showed consistent lower performance across models. Both datasets exhibited low recall and ROC AUC scores, which suggests possible negative influence on generalization. The exclusion of these datasets was motivated by their likely distributional mismatch, potential annotation inconsistencies, or image quality issues that introduced noise into the training process.

Table 9 presents the evaluation results of several model architectures employing the Merged Dual-View (MDV) approach under this refined setting, referred to as Setting 2 (Figure 7).

The updated setting has led to better performance metrics. In particular, F1 scores and ROC AUC values improved for almost all architectures, suggesting better class balance and improved discriminative ability. Notably, the top-performing models—VGG19 and DenseNet—achieved particularly strong results.

DenseNet showed the most balanced and consistent results, achieving an F1 score of 0.7582 and a ROC AUC of 0.7960. Its high precision (0.7489) and recall (0.7677) reflect strong predictive power for both positive and negative cases, underscoring DenseNet’s architectural suitability for complex, high-resolution medical imaging analysis.

Another significant performer was the Vision Transformer (ViT-B/16), which demonstrated notable sensitivity to positive cases with a recall score of 0.9013, the highest among all models. Correct identification of the majority of positive cases is a very important characteristic in medical settings where there are severe consequences for missing positive cases. ViT-B/16 also achieved a competitive F1 score of 0.7728, despite slightly lower precision.

In contrast, MobileNet, while achieving relatively high accuracy (0.7929), showed poor performance on other key metrics, particularly precision (0.3371), recall (0.1432), and F1 score (0.2010). The significant discrepancy between accuracy and F1 score indicates that the model likely overfits to dominant classes or exhibits poor generalization to minority classes.

ResNet18, a baseline CNN architecture, also benefited from the dataset refinement, achieving a respectable F1 score of 0.6776 and a ROC AUC of 0.7434. These results represent a substantial improvement compared to its prior performance when all datasets were included, indicating that data quality and homogeneity play a crucial role in model learning dynamics.

In summary, this experiment highlights the significant impact of dataset curation on model performance. By excluding noisy datasets, the MDV approach becomes more effective at capturing reliable features and generalizing across clean domains. These results demonstrate that careful dataset selection is not only beneficial but perhaps necessary in multi-source medical AI development. The findings further suggest that architectural advantages (e.g., DenseNet’s connectivity or ViT’s global attention) are best realized when training data is coherent and of sufficient quality.

### 4.4. Performance of the Dual-Branch Ensemble (DBE) Method

This experiment continues with refined evaluation strategy outlined in Setting 2 (Figure 7), which excludes the NLBS and CSAW-CC datasets. The Dual-Branch Ensemble (DBE) method was applied to this curated dataset split to evaluate its effectiveness across a range of model backbones.

Table 10 presents the performance metrics of various DBE configurations. DenseNet emerged as the top performer, achieving the highest F1 score (0.7756) and one of the highest ROC AUC values (0.8033). This result indicates that DenseNet, when used within the DBE framework, maintains a strong balance between sensitivity and precision.

ResNet50 and VGG19 also produced robust results. ResNet50 reached an F1 score of 0.7328 and the highest ROC AUC overall (0.8042), confirming its capacity for high-fidelity feature extraction and reliable decision making in ensemble-based architectures. VGG19 followed closely, with a strong recall of 0.8216 and an F1 score of 0.7574, demonstrating its effectiveness in identifying positive cases even in relatively smaller or imbalanced datasets. These results affirm the general compatibility of DBE with traditional convolutional backbones.

An especially notable outcome was observed with MobileNet, which performed significantly better under the DBE configuration compared to its results in the MDV setting. Its F1 score increased to 0.7147 and ROC AUC to 0.7757, a substantial improvement over its previous metrics. This suggests that DBE’s architecture can offset some of the limitations associated with lightweight models like MobileNet, particularly their reduced representational capacity.

On the other hand, ViT-B/16 underperformed relative to all other models. It achieved the lowest F1 score (0.5700) and ROC AUC (0.5696), indicating architectural misalignment with the DBE framework. This result may be attributed to the ViT’s lack of inductive bias toward locality and its difficulty in capturing multi-scale features across heterogeneous views without large amounts of training data. Additionally, the DBE fusion mechanism may not integrate well with the ViT’s patch-based token representations, leading to suboptimal learning dynamics.

**Figure 10:**
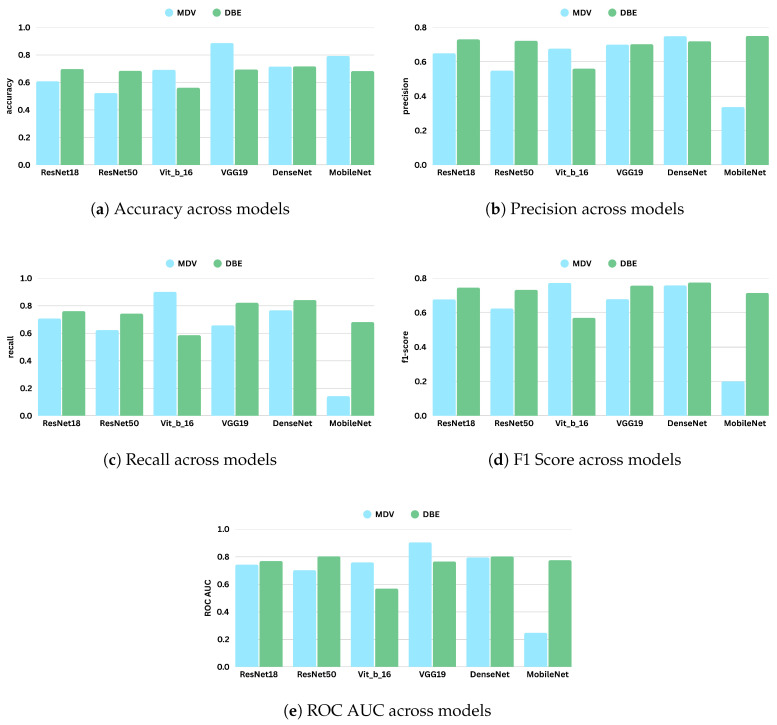
Performance comparison of different models under the Merged Dual-View (MDV) and Dual-Branch Ensemble (DBE) frameworks based on evaluation metrics: accuracy, precision, recall, F1 score, and ROC AUC (Setting 2). Each subfigure shows a bar chart comparing models across the two frameworks for a specific metric.

**Figure 11:**
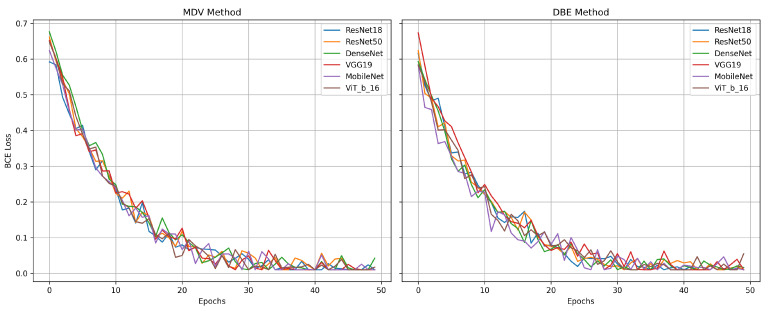
Validation loss for MDV and DBE methods under Setting 2.

Overall, the results from this experiment reinforce the effectiveness of the DBE method, particularly when applied to curated and consistent datasets. By maintaining separate processing paths for CC and MLO views, DBE leverages the unique diagnostic content of each view more effectively than merged-input models. This architectural choice enables deeper models such as DenseNet and ResNet50 to learn complementary representations, yielding improved classification outcomes. Figure 10 represents the comparative diagrams for DBE and MDV methods across models and metrics. Figure 11 represents the validation losses.

These findings support the continued use and refinement of ensemble-based architectures in mammography CAD systems, especially when model robustness and generalization are critical deployment factors.

## 5. Discussion

The comparative results showed that both MDV and DBE approaches outperformed single-view baselines, underscoring the value of dual-view integration. The MDV strategy, which spatially merges CC and MLO views into a single image, performed well with models such as VGG19 and DenseNet. These architectures effectively learned fused spatial representations and captured inter-view correlations. However, the DBE approach—where CC and MLO views are processed independently by two dedicated CNN branches before combining their outputs—exhibited stronger and more stable generalization, especially when applied to heterogeneous datasets. DenseNet and ResNet50 demonstrated particularly robust behavior under the DBE setting, suggesting that separate feature learning followed by ensemble fusion can better preserve view-specific information and mitigate noise introduced by misaligned or low-quality images.

Despite these advancements, the classification metrics reported in this study are more conservative compared to those in earlier literature. This discrepancy likely stems from differences in evaluation protocols. Many prior studies relied on single curated datasets and report performance in controlled environments with minimal variation. In contrast, our work employed a challenging cross-domain evaluation using multiple datasets that reflect real-world variability in patient demographics, imaging hardware, and acquisition quality. These conditions expose the vulnerabilities of deep models to domain shift, but they also offer a more realistic assessment of AI readiness for clinical deployment.

Model performance improved notably when we excluded low-quality or distributionally mismatched datasets, indicating the sensitivity of deep learning models to training data quality. Under this refined configuration, the DBE approach with DenseNet achieved an F1 score of 0.7756 and an AUC of 0.8033, while VGG19 and ResNet50 also demonstrated competitive results. Interestingly, the vision transformer (ViT), while achieving high recall in the MDV setting (0.9013), underperformed when applied within the DBE framework. This highlights an important architectural consideration: transformer-based models, which rely on global attention mechanisms and patch-level embeddings, may not generalize well when views are processed independently and merged at the decision level. These results suggest that ViTs may require specialized fusion strategies—such as cross-attention or shared token spaces—to effectively capture inter-view relationships.

**Figure 12:**
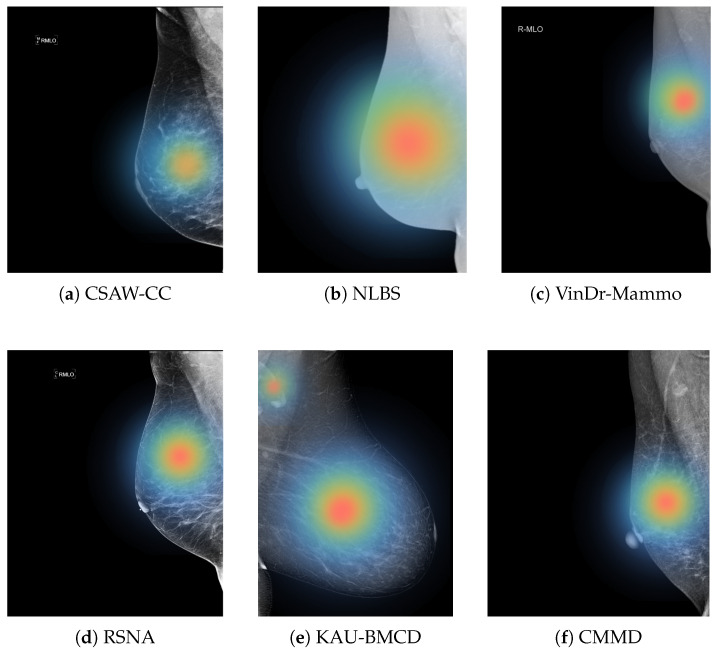
Examples of heat maps for each dataset.

Another key finding is the need for future studies to move beyond standalone model evaluation and investigate how AI systems can support or augment radiologist decision-making. While high AUC and recall scores are promising, they do not directly reflect real-world utility. Clinical integration requires understanding how model outputs influence radiologist sensitivity, specificity, and diagnostic confidence. Empirical studies involving radiologists with and without AI assistance will be necessary to assess true clinical value and to refine model behavior based on human–AI interaction. Figure 12 illustrates regions that the model considers important for its classification, which can potentially help radiologists focus on subtle or ambiguous areas that may otherwise be overlooked.

In summary, this work contributes to the development of clinically relevant AI systems for mammography by exploring multi-view fusion strategies and rigorously testing model generalization across diverse imaging conditions. The results demonstrate that both architectural choices and dataset curation significantly influence model performance. Further research should explore domain-invariant feature learning, transformer-based multi-view integration, and hybrid systems that combine imaging data with metadata or radiology reports. These directions will be essential for translating algorithmic performance into practical, trustworthy tools for early breast cancer detection.

## 6. Conclusions

This study advances the development of AI-assisted mammography by investigating multiple view integration strategies for breast cancer classification. By incorporating six diverse mammography datasets representing different institutions, imaging systems, and populations, we emphasized the importance of dataset diversity in building robust and clinically relevant diagnostic models.

Our findings demonstrate that leveraging both CC and MLO views leads to significantly improved classification performance compared to single-view approaches. In particular, the DBE method, which processes each view independently before fusion, achieved superior results with architectures such as DenseNet and ResNet50.

However, performance varied notably between seen and unseen datasets, highlighting the persistent challenge of domain shift. Models that performed well on familiar data often failed to generalize to external datasets, underscoring the fragility of current deep learning approaches in heterogeneous clinical environments. By excluding lower-quality or distributionally mismatched datasets, we observed improved performance and consistency across models.

Improving generalizability remains a key challenge. Future research should explore domain-invariant feature learning, self-supervised pretraining, and meta-learning techniques to mitigate distributional gaps across datasets. Additionally, integrating non-image modalities such as radiology reports, patient metadata, and prior imaging can help build context-aware models that align more closely with clinical decision making.

Although this work focused on algorithmic performance, future directions must also address model interpretability and human–AI collaboration. With further development, saliency maps and attention visualizations could serve as assistive tools for radiologists, enhancing diagnostic confidence and efficiency. Ultimately, prospective validation in clinical workflows will be essential to realize the full potential of AI in mammography.

In conclusion, our study provides a robust foundation for the development of generalizable, interpretable, and clinically deployable AI systems for breast cancer screening. By advancing multi-view fusion and cross-domain evaluation, we move one step closer to safe and effective integration of AI in routine diagnostic practice.

## Figures and Tables

**Table 1:** Overview of datasets used in the cross-dataset evaluation experiment.

Dataset	Total Images	Normal	Abnormal	Modality	Machine Type	Institution Type	Region
VinDr-Mammo	20,000	13,406	6594	FFDM	Hologic	Hospital-based screening	Vietnam
CMMD	3734	0	3734	FFDM	GE Senograph	Cancer center	China
CSAW-CC	82,676	79,984	2692	FFDM	Hologic	Population screening	Sweden
NLBS	26,988	20,257	6731	FFDM	GE Senograph	Population screening	Canada
KAU-BCMD	2180	1824	356	FFDM	IMS Giotto	University hospital	Saudi Arabia
RSNA	47,652	45,224	2428	FFDM + CR	Hologic, GE, Siemens	Multi-institutional	-

**Table 2:** Dataset statistics after undersampling.

Dataset	Train Normal	Train Abnormal	Test Normal	Test Abnormal
VinDr	2916	5308	772	1286
CMMD	0	2986	0	748
CSAW-CC	5010	2172	1276	520
KAU-BCMD	408	280	96	76
NLBS	5042	5044	1028	524
RSNA	4304	1904	1262	1260

**Table 3:** Performance of MDV models across the datasets—unseen patients.

Model	Unseen Dataset Name	Accuracy	Precision	Recall	F1	ROC AUC
ResNet18	NLBS	0.8255	0.3567	0.1896	0.2476	0.6209
ResNet18	VinDr-Mammo	0.5447	0.4538	0.5100	0.4803	0.5491
ResNet18	CSAW-CC	0.4294	0.1580	0.6842	0.2568	0.5674
ResNet18	KAU-BCMD	0.6034	0.2865	0.5506	0.3769	0.63701
ResNet18	RSNA	0.4836	0.1584	0.5568	0.2466	0.5257

**Table 4:** Performance of MDV models across the datasets—seen patients.

Model	Seen Dataset Name	Accuracy	Precision	Recall	F1	ROC AUC
ResNet18	NLBS	0.4970	0.2661	0.5010	0.3470	0.3969
ResNet18	VinDr-Mammo	0.5675	0.6937	0.5833	0.6337	0.5712
ResNet18	CSAW-CC	0.5401	0.3548	0.6543	0.4601	0.6197
ResNet18	KAU-BCMD	0.6628	0.5714	0.7778	0.6588	0.7644
ResNet18	RSNA	0.5760	0.3909	0.6337	0.4835	0.6184

**Table 5:** Performance of DBE models across the datasets—unseen patients.

Model	Unseen Dataset Name	Accuracy	Precision	Recall	F1	ROC AUC
ResNet18	NLBS	0.5110	0.5418	0.1427	0.2259	0.5145
ResNet18	VinDr-Mammo	0.5944	0.7407	0.0243	0.0470	0.5308
ResNet18	CSAW-CC	0.6443	0.1690	0.3752	0.2330	0.5492
ResNet18	KAU-BCMD	0.7870	0.6000	0.0674	0.1212	0.6241
ResNet18	RSNA	0.6253	0.1047	0.1944	0.1361	0.4368

**Table 6:** Performance of DBE models across the datasets—seen patients.

Model	Seen Dataset Name	Accuracy	Precision	Recall	F1	ROC AUC
ResNet18	NLBS	0.5400	0.5353	0.6016	0.5665	0.5695
ResNet18	VinDr-Mammo	0.5909	0.6853	0.6697	0.6774	0.5850
ResNet18	CSAW-CC	0.7004	0.5000	0.3829	0.4337	0.6766
ResNet18	KAU-BCMD	0.6395	0.5455	0.8333	0.6593	0.8178
ResNet18	RSNA	0.6740	0.4779	0.4444	0.4606	0.6697

**Table 7:** Performance of the Merged Dual-View (MDV) model across multiple mammography datasets.

Model	Dataset	Accuracy	Precision	Recall	F1	ROC AUC
ResNet18	RSNA	0.5967	0.5719	0.6737	0.6187	0.6822
ResNet18	KAU-BCMD	0.8165	0.4130	0.5938	0.4872	0.5577
ResNet18	VinDr	0.6910	0.5582	0.4501	0.4984	0.5555
ResNet18	NLBS	0.6565	0.2898	0.1927	0.2315	0.2740
ResNet18	CSAW-CC	0.8440	0.4140	0.2680	0.3250	0.3806

**Table 8:** Performance of the Dual-Branch Ensemble (DBE) model across multiple mammography datasets.

Model	Dataset	Accuracy	Precision	Recall	F1	ROC AUC
ResNet18	RSNA	0.5967	0.5746	0.6525	0.6111	0.6339
ResNet18	KAU-BCMD	0.8853	0.6400	0.5000	0.5614	0.9283
ResNet18	VinDr	0.6632	0.7127	0.2921	0.4144	0.6578
ResNet18	NLBS	0.5765	0.5745	0.5873	0.5808	0.6050
ResNet18	CSAW-CC	0.8625	0.5465	0.1801	0.2709	0.7235

**Table 9:** Performance of the Merged Dual-View (MDV) method under Setting 2 (Figure 7).

Model Name	Accuracy	Precision	Recall	F1	ROC AUC
ResNet18	0.6084	0.6499	0.7077	0.6776	0.7434
ResNet50	0.5234	0.5486	0.6236	0.6237	0.7030
Vit_b_16	0.6918	0.6764	0.9013	0.7728	0.7600
VGG19	0.8865	0.7003	0.6578	0.6783	0.9051
DenseNet	0.7152	0.7489	0.7677	0.7582	0.7960
MobileNet	0.7929	0.3371	0.1432	0.2010	0.2478

**Table 10:** Performance of Dual-Branch Ensemble (DBE) models under Setting 2 (Figure 7).

Model Name	Accuracy	Precision	Recall	F1	ROC AUC
ResNet18	0.6980	0.7305	0.7616	0.7457	0.7700
ResNet50	0.6848	0.7225	0.7434	0.7328	0.8042
VGG19	0.6940	0.7026	0.8216	0.7574	0.7667
MobileNet	0.6834	0.7508	0.6819	0.7147	0.7757
Vit_b_16	0.5622	0.5600	0.5863	0.5700	0.5696
DenseNet	0.7170	0.7195	0.8413	0.7756	0.8033

## Data Availability

Dataset available on open access: KAU-BCMD: https://www.kaggle.com/datasets/orvile/kau-bcmd-mamography-dataset (accessed on 10 January 2025); RSNA: https://www.kaggle.com/competitions/rsna-breast-cancer-detection (accessed on 10 January 2025); CMMD: https://www.kaggle.com/datasets/tommyngx/cmmd2022 (accessed on 10 January 2025). Dataset available on request: VinDr-Mammo: https://vindr.ai/datasets/mammo (accessed on 10 January 2025); NLBS: https://www.frdr-dfdr.ca/repo/dataset/cb5ddb98-ccdf-455c-886c-c9750a8c34c2 (accessed on 20 January 2025); CSAW-CC: https://gts.ai/dataset-download/csaw-cc-mammography/ (accessed on 25 January 2025).

## Abbreviations

The following abbreviations are used in this manuscript:

CNN	Convolutional Neural Network
DL	Deep Learning
CC	Craniocaudal
MLO	Mediolateral oblique
